# Mönckeberg calcified sclerosis preventing the use of radial artery in myocardial revascularization surgery

**DOI:** 10.4322/acr.2024.513

**Published:** 2024-09-06

**Authors:** Luiz Alberto Benvenuti

**Affiliations:** 1 Universidade de São Paulo (USP), Faculdade de Medicina, Hospital das Clínicas, Instituto do Coração (InCor), São Paulo, SP, Brasil

**Keywords:** Arteriosclerosis, Coronary Artery Bypass, Mönckeberg Medial Calcific Sclerosis, Radial Artery

**Figure 1:**
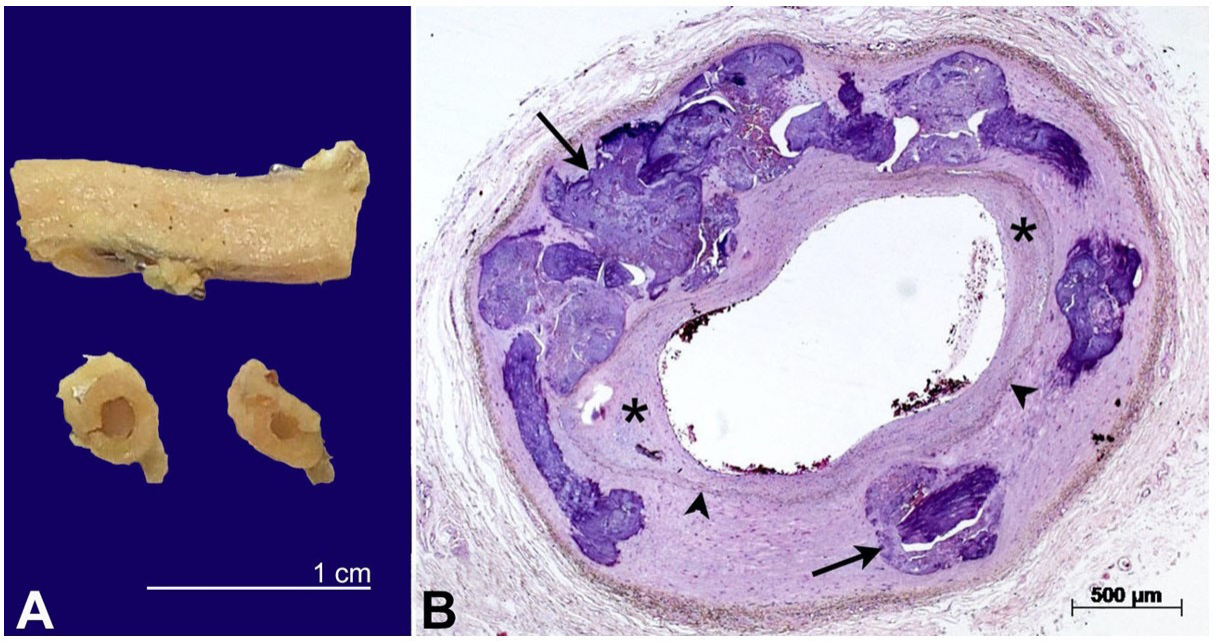
**A –** external and cross-sectional macroscopic view of the radial artery. Note thickening of the arterial wall; **B –** at histology, massive media calcification is evident (arrows), plus mild intimal fibrotic thickening (asterisks). Note internal elastic lamina (arrowheads), the internal limit of the medial layer (HE stain).

Arteriosclerosis, i.e., “hardening of the arteries”, is currently subdivided into atherosclerosis, Mönckeberg calcified sclerosis (MCS), and arteriolosclerosis. MCS was first described in 1903, characterized by calcific deposits affecting only the tunica media of large and medium-sized muscular arteries without significant luminal stenosis. However, MCS can coexist with classical atherosclerosis, characterized by atheroma plaques in the intima. MCS is rarely seen in patients under the 5^th^ decade of life and is associated with the presence of diabetes mellitus and/or chronic kidney disease. The etiology and pathogenesis of MCS are currently poorly understood.^1,2^

The use of arterial grafts in myocardial revascularization surgery is associated with a lower rate of adverse cardiac events and a higher rate of patency of the grafts at 5 years of follow-up. Besides the left internal thoracic artery, the right one and the radial artery have been used, particularly when complete revascularization is pursued in patients with multivessel coronary artery disease.^3^

However, when planning myocardial revascularization, surgeons should be aware that pathological alterations, particularly arteriosclerosis, can affect arterial conduits. Concerning the radial artery, there are previous reports of MCS preventing arterial catheterization and, more importantly, preventing its use as an arterial graft in myocardial revascularization surgery.^4,5^

A 69-year-old man presented to our hospital with unstable angina. He had systemic hypertension and type II insulin-dependent diabetes mellitus. A previous myocardial infarction occurred 25 years ago, treated with coronary angioplasty and a stent position in the right coronary artery. He was previously submitted to amputation of several toes. No renal dysfunction was detected during the current hospitalization. Coronary angiography demonstrated severe obstructive atherosclerotic lesions in the left trunk and anterior descending coronary artery, plus occlusive thrombosis of the right coronary artery. Myocardial revascularization surgery was planned with the use of internal thoracic and radial arteries. However, at the operation table, the surgeon noticed the radial artery was severe and diffusely calcified and decided to abort its use. The revascularization surgery was successfully concluded using the left internal thoracic artery and two saphenous vein grafts.

The Pathology laboratory received a 16 cm long artery presenting an external diameter ranging from 0.4 to 0.6 cm. The artery was diffusely hardened and presented a calcified aspect. After chemical decalcification, the artery was cross-sectioned and moderate to severe thickening of the wall was noticed (Figure 1A). At histology, there was massive calcification of the media and mild multifocal, fibrotic thickening of the intima (Figure 1B). There were no lipid deposits or calcification of the intima. We concluded for severe MCS plus mild atherosclerosis.
